# Supplementary material for: Pregelatinized green banana flour snack bars: Physicochemical properties, sensory quality, and gastroprotective potential in an ethanol-induced ulcer model
Source: PLoS One. 2026 Apr 24;21(4):e0347771. doi: 10.1371/journal.pone.0347771 (PMC13108814; doi:10.1371/journal.pone.0347771)
Supplement: Attachment — Submitted filename: Respond to reviewer (point-by-point) Revised_(Gastric ulcer).pdf [file pone.0347771.s003.pdf]

School of Health Science,
Mae Fah Luang University

27th December 2025

Editor-in-Chief
PLOS ONE

Dear Editors,

On behalf of my co-authors, I am pleased to resubmit our revised manuscript titled: "Pregelatinized Green Banana Flour Snack Bars: Physicochemical Properties, Sensory Quality, and Gastroprotective Potential in an Ethanol-Induced Ulcer Model." (Manuscript ID: PONE-D-25-49266) for reconsideration for publication in PLOS ONE. We would like to express our sincere gratitude to the editorial team and reviewers for their thoughtful and constructive feedback. We have thoroughly addressed all comments and suggestions in the revised manuscript and the accompanying point-by-point response document, as listed at the end of the cover letter.

We have also fully complied with PLOS ONE's journal-specific requirements, including reformatting the manuscript according to PLOS style templates and correcting the funding statement to accurately reflect the role of each funding source. We would like to declare the roles of each financial support as follows. The materials testing in this manuscript has been developed and supported by the research that received financial support from the National Science, Research, and Innovation Fund (NSRF) and Mae Fah Luang University (Fundamental Fund Grant No. 662A05032). The animal experiment was supported by the Southern Thailand Science Park (Collaborative Research Program; Co-Research-PsuCor-1/62), Prince of Songkla University. The manuscript's production was partially assisted through academic collaboration under the reinventing university system of Mae Fah Luang University, which helped in bridging the international team to work together. Furthermore, the article processing charge (APC) is supported by Mae Fah Luang University. Therefore, we would like to revise the funding statement as follows. "The research received financial support from the National Science, Research, and Innovation Fund (NSRF) and Mae Fah Luang University (Fundamental Fund Grant No. 662A05032). It was also partly supported by the Southern Thailand Science Park (Collaborative Research Program; Co-Research-PsuCor-1/62), Prince of Songkla University. The manuscript's production was partially supported through academic collaboration within the reinventing university system at Mae Fah Luang University. The article processing charge (APC) is supported by Mae Fah Luang University. The funders had no role in the study design, data collection and analysis, the decision to publish, or the preparation of the manuscript."

We believe that the revised version significantly improves the clarity, methodological transparency, and scientific rigor of our work, aligning with PLOS ONE's editorial standards and scope. I appreciate your consideration and look forward to your favorable response.

Best Regards,

Sasitorn Chusri, Ph.D.
Corresponding Author
Biomedical Technology Research Group for Vulnerable Populations,
School of Health Science, Mae Fah Luang University
Muang, Chiang Rai 57100, Thailand
E-mail: sasitorn.chu@mfu.ac.th

Journal Requirements:

When submitting your revision, we need you to address these additional requirements.

1. Please ensure that your manuscript meets PLOS ONE's style requirements, including those for file naming. The PLOS ONE style templates can be found at

https://journals.plos.org/plosone/s/file?id=wjVg/PLOSOne_formatting_sample_main_body.pdf and https://journals.plos.org/plosone/s/file?id=ba62/PLOSOne_formatting_sample_title_authors_affiliations.pdf

Address the journal's comment: Thank you very much for the suggestion. The author has proofread the revised manuscript and confirms that it meets PLOS ONE's style requirements, as mentioned.

2. Please include your tables as part of your main manuscript and remove the individual files. Please note that supplementary tables (should remain/ be uploaded) as separate "supporting information" files
Address the journal's comment: Thank you for pointing this out for us. The author has included the tables in the revised manuscript, as requested.

3. Thank you for stating the following financial disclosure: [The research received financial support from the National Science, Research, and Innovation Fund (NSRF) and Mae Fah Luang University (Fundamental Fund Grant No. 662A05032). It was also partly supported by the Southern Thailand Science Park (Collaborative Research Program; Co-Research-PsuCor-1/62), Prince of Songkla University. The manuscript's production was partially assisted through academic collaboration under the reinventing university system of Mae Fah Luang University.]. Please state what role the funders took in the study. If the funders had no role, please state: "The funders had no role in study design, data collection and analysis, decision to publish, or preparation of the manuscript." If this statement is not correct you must amend it as needed.
Please include this amended Role of Funder statement in your cover letter; we will change the online submission form on your behalf.

Address the journal's comment: Thank you for your kind concerns. We would like to declare the roles of each financial support as follows. The materials testing in this manuscript has been developed and supported by the research that received financial support from the National Science, Research, and Innovation Fund (NSRF) and Mae Fah Luang University (Fundamental Fund Grant No. 662A05032). The animal experiment was supported by the Southern Thailand Science Park (Collaborative Research Program; Co-Research-PsuCor-1/62), Prince of Songkla University. The manuscript's production was partially supported through academic collaboration under the reinventing university system at Mae Fah Luang University, which helped the international team work together. Furthermore, the article processing charge (APC) is supported by Mae Fah Luang University. Therefore, we would like to revise the funding statement as follows.

[The research received financial support from the National Science, Research, and Innovation Fund (NSRF) and Mae Fah Luang University (Fundamental Fund Grant No. 662A05032). It was also partly supported by the Southern Thailand Science Park (Collaborative Research Program; Co-Research-PsuCor-1/62), Prince of Songkla University. The manuscript's production was partially assisted through academic collaboration under the reinventing university system of Mae Fah Luang University. The article processing charge (APC) is supported by Mae Fah Luang University.]

4. We note that your Data Availability Statement is currently as follows: [All relevant data are within the manuscript and its Supporting Information files]

Please confirm at this time whether or not your submission contains all raw data required to replicate the results of your study. Authors must share the "minimal data set" for their submission. PLOS defines the minimal data set to consist of the data required to replicate all study findings reported in the article, as well as related metadata and methods (https://journals.plos.org/plosone/s/data-availability#loc-minimal-data-set-definition).

For example, authors should submit the following data:

- The values behind the means, standard deviations and other measures reported;
- The values used to build graphs;
- The points extracted from images for analysis.

Authors do not need to submit their entire data set if only a portion of the data was used in the reported study. If your submission does not contain these data, please either upload them as Supporting Information files or deposit them to a stable, public repository and provide us with the relevant URLs, DOIs, or accession numbers. For a list of recommended repositories, please see https://journals.plos.org/plosone/s/recommended-repositories.

If there are ethical or legal restrictions on sharing a de-identified data set, please explain them in detail (e.g., data contain potentially sensitive information, data are owned by a third-party organization, etc.) and who has imposed them (e.g., an ethics committee). Please also provide contact information for a data access committee, ethics committee, or other institutional body to which data requests may be sent. If data are owned by a third party, please indicate how others may request data access.

Address the journal's comment: The information including the raw data used in this manuscript was provided in the Supporting Information files under the name 'Final Report'.

5. Please include a separate caption for each figure in your manuscript.
Address the journal's comment: The caption for each figure was included in the revised manuscript.

6. Please include a copy of Table 1-4 which you refer to in your text on page10, 11 and 13.
Address the journal's comment: The Table 1-4 was included in the revised manuscript.

7. If the reviewer comments include a recommendation to cite specific previously published works, please review and evaluate these publications to determine whether they are relevant and should be cited. There is no requirement to cite these works unless the editor has indicated otherwise.
Address the journal's comment: Thank you very much for your concerns, there is no recommendation to cite specific previously published works from the reviewers.

Reviewer #1: Overall assessment: This is a well-designed and comprehensive study that investigates the development of a functional food product with a clear therapeutic target. The manuscript is generally well-written, the methodology is sound, and the results support the conclusions. The work has significant potential for practical application in the functional food industry. However, there are areas where clarity, data presentation, and statistical rigor can be improved to strengthen the manuscript for publication.

A. Inconsistencies and points of confusion

1. Dosage Form in Animal Study: The animal study administers "flour" (GBF and PregelF3) at 1000 mg/kg. Administering a dry powder to rats via oral gavage is technically challenging and can cause aspiration. It is standard practice to suspend or dissolve the test substance in a vehicle (e.g., water, carboxymethyl cellulose). The manuscript must clarify how the flours were administered.

Address the reviewer's comment:

Thank you for highlighting this important methodological detail. We agree that directly administering dry powder is not practical for oral gavage and may pose a risk to the animals. We have now clarified the administration procedure in the Materials and Methods (Section 2.4) as follows:

"Both GBF and PregelF3 powders were freshly suspended in distilled water immediately before dosing to obtain a homogeneous slurry suitable for oral administration. The suspensions were administered to the rats by oral gavage at a dose of 10 mL/kg body weight to ensure consistent delivery of the 1000 mg/kg dose. This method ensured accurate dosing, minimized the risk of aspiration, and complied with standard procedures for safe oral administration in rodents."

This revision specifies the vehicle used, the preparation method, and ensures compliance with standard gavage safety practices. Thanks again for emphasizing this point and for helping to clarify our methodology.

2. Statistical Analysis Presentation: While ANOVA is mentioned, the post-hoc test (Duncan's) is not always clearly applied in the results text. For example, in Table 3, some values are described as "significantly" different without the corresponding letters being referenced in the narrative. The figures (Fig. 3) lack statistical annotation (e.g., letters or asterisks) to show which groups are significantly different from each other.

Address the reviewer's comment: Thank you very much for your feedback. All tables and figure legends now reference consistent superscript letters. Figure 3 now includes asterisks and pound signs indicating statistical differences (* vs. vehicle; # vs. sham), and as a result, the text in Results was revised for accuracy.

3. Sensory Evaluation Scale: The manuscript states a "9-point hedonic scale" is used but does not define the anchors (e.g., 1=dislike extremely, 9=like extremely). This is a minor but important detail for reproducibility.

Address the reviewer's comment: The information was added in the Methods section as follows.

"The 9-point hedonic scale was anchored as follows: 1 = dislike extremely, 2 = dislike very much, 3 = dislike moderately, 4 = dislike slightly, 5 = neither like nor dislike, 6 = like slightly, 7 = like moderately, 8 = like very much, and 9 = like extremely. "

4. Microbiological Data in Table 4: The table lists "ND" for Total Viable Count (TVC) at week 0 but `1.1x10^2` CFU/g at week 2. An increase from ND to 10^2 is unusual for a baked, low-moisture product and should be briefly explained (e.g., potential for post-baking contamination during handling, or clarification that "ND" means below a certain detection limit, like <10 CFU/g).

Address the reviewer's comment: Thank you very much, we added clarification in the Results and Table 3:

An acceptable microbiological quality was also achieved in the formulated snack bar, with total viable microbial counts stabilizing around 10^2 CFU/g after the second week. In comparison, yeast and mold counts remained generally low (<10 CFU/g). At week 0, total viable counts were below the detection limit (<10 CFU/g). The slight increase to approximately 10^2 CFU/g observed by week 2 may be attributed to minor post-baking contamination during handling or packaging.

B. Section-by-section analysis with recommendations
ABSTRACT

Line 4-5: "well-recognized gluten-free flour" – This is true, but not the primary focus. Consider rephrasing to emphasize its recognized gastroprotective potential in traditional medicine/preclinical studies.

Line 10-11: "systematically investigated" – Good phrasing, it accurately reflects the study's strength.

Address the reviewer's comment: Thank you very much for your kind suggestion and encouraging our research. The abstract was revised by rephrasing the opening sentence, adding a clear objective statement, and including numerical results. Additionally, the keywords have been updated to be more

specific: gastroprotection; pregelatinized banana flour; resistant starch; functional food; ethanol-induced ulcer model; snack bar formulation.

INTRODUCTION

Line 34-35: "However, in addition to this widespread ethnobotanical use, there is no evidence of its gastroprotective effects." – This statement is incorrect and a major error. The sentences immediately preceding this (Lines 30-33) cite references [13, 14, 15] that provide evidence of gastroprotective effects. This line must be deleted or heavily rephrased (e.g., "However, evidence for the specific cultivar Musa sapientum Linn., ABB group, cv. Kluai Namwa, used in Thai medicine is limited").

Line 41-42: "may also influence bioactivity" – This is a key point of your hypothesis. You can state this more confidently, e.g., "but its impact on bioactivity, particularly gastrointestinal protective potential, remains unexplored."

Address the reviewer's comment: Thank you very much for the very informative suggestion. The introduction section was revised to remove the incorrect claim that no gastroprotective evidence exists, to indicate the gap in knowledge, and to strengthen the rationale and novelty. The following information was added.

"Among them, the unripe Musa species used to make green banana flour (GBF) can be singled out as a highly nutritious source of resistant starch, phenolic antioxidants, and non-digestible fiber [8-9]. Its gastroprotective properties have been demonstrated in several preclinical studies, which show reductions in gastric and intestinal inflammation across various models [10-15]. Moreover, its long-range ethnopharmacological history of use in traditional Thai and Sudanese medicine for the treatment of peptic ulcer disease has long been regarded as evidence of its therapeutic potential [13,16].

Nonetheless, there are challenges associated with the direct application of native GBF of food formulations. Its low solubility, roughness, and low digestibility limit its sensory properties and industrial application [17,18]."…

"Although these advantages are well known, a research gap remains regarding the effects of pregelatinization on the gastroprotective activity of GBF and its functional properties in consumer-acceptable edible foods. The study was thus aimed at determining the physicochemical properties of pregelatinized drum-dried GBF, comparing its gastroprotective effects with those of native GBF using an ethanol-induced gastric ulcer rat model, and evaluating shelf-stable snack bars containing pregelatinized GBF in terms of their sensory and storage properties.

We posit that pregelatinization does not alter the gastroprotective performance of GBF but improves its functional and physicochemical characteristics, thereby developing a palatable, stable, health-promoting snack bar that can be used in gastrointestinal nutritional support."

MATERIALS AND METHODS

Section 2.1: Line 68-70: Specify the concentration of GBF in the slurry for each formulation (PregelF1-F4) here, or state that they are detailed in Table 1. It's currently in Table 1, but mentioning the range (20-50%) in the text improves flow.

Address the reviewer's comment: The concentration of GBF was specified as recommended in Section 2.1.

‘The study employed a completely randomized design (CRD) to investigate the effects of GBF concentrations (20%, 30%, 40%, and 50% w/v) on the physicochemical properties of drum-dried formulations designated PregelF1, PregelF2, PregelF3, and PregelF4, respectively.’

Section 2.2: Line 80-81: "using the published protocol" – This is too vague. You must cite the specific protocol or describe the method briefly (which you do in the following sentences). Delete "using the published protocol."

Address the reviewer’s comment: The text was deleted as suggested. The specific protocol was cited as mentioned.

Section 2.4 (Gastroprotective effects): Line 125-126: Crucial Omission. You must describe how the GBF and PregelF3 powders were prepared for oral administration to the rats. Were they suspended in water or another vehicle? This is a critical methodological detail.

Address the reviewer’s comment: The information has been added to Section 2.6, Gastroprotective effects, as follows.

“Both GBF and PregelF3 powders were freshly suspended in distilled water immediately before dosing to obtain a homogeneous slurry suitable for oral administration. The suspensions were administered to the rats by oral gavage at a dose of 10 mL/kg body weight to ensure consistent delivery of the 1000 mg/kg dose. This method ensured accurate dosing, minimized the risk of aspiration, and complied with standard procedures for safe oral administration in rodents.”

Line 143: "Gastric contents were collected to measure pH" – Specify how the gastric contents were collected (e.g., by gentle scraping or washing with a fixed volume of water?).

Address the reviewer’s comment: The section has been revised for greater clarity, as follows.

“After the stomachs were excised and opened along the greater curvature, the gastric contents were gently collected by rinsing the stomach lumen with a fixed volume of distilled water (2 mL) using a sterile Pasteur pipette into pre-labelled tubes. The pH of each sample was immediately measured using a calibrated pH meter (Thermo Fisher Scientific, MA, USA).”

RESULTS

Table 1: The footnote says "Values are expressed as mean±SD (n=3)." This is good. Ensure all values in the table carry the same number of decimal places for consistency (e.g., Water Activity is given to 3 decimal places, which is fine, but be consistent).

Address the reviewer’s comment: Thank you for this helpful suggestion. We agree that consistent numerical formatting enhances data clarity and presentation quality. We have updated Table 1 to ensure that all reported values are shown with a consistent number of decimal places within each parameter (e.g., water activity reported consistently to three decimal places, moisture content to two decimal places, and color parameters to two decimal places). This revision improves readability and aligns with good scientific reporting standards. We appreciate the reviewer’s attention to detail, which has contributed to improving the overall quality of the manuscript.

Yield: It would be helpful to briefly speculate in the text on why yield decreases with higher GBF concentration (e.g., increased viscosity leading to more adhesion to the drums).

Address the reviewer's comment: Thank you for this insightful suggestion. We agree that a brief explanation enhances the interpretation of the yield data. Accordingly, we have added the following explanation to the Results section (Section 3.1, Physicochemical properties of pregelatinized green banana flour).

"Increasing GBF concentrations (20–50% w/v) resulted in a gradual reduction in yield from 88.9% (PregelF1) to 86.9% (PregelF4). The decline in yield at higher concentrations is likely due to increased viscosity and stickiness of the GBF slurry, which promotes greater adhesion to the drum surface during drying and thereby reduces product recovery. This indicates that higher GBF concentrations could reduce processing efficiency, likely due to increased viscosity or adhesion during drum drying."

Table 2 & Sensory Evaluation: The text states sPreF3 had the highest ratings, but the statistical analysis (letters) in Table 2 shows that for most attributes (Color, Flavor, Taste, Overall Appearance), sPreF3 is not significantly different from other formulations. The narrative should be tempered to reflect this, e.g., "sPreF3 received numerically higher ratings... and was significantly preferred for crunchiness over sPreF4."

Address the reviewer's comment: Thank you for this valuable comment. We agree that the narrative should accurately reflect the statistical outcomes presented in Table 2. We have revised the Results and Discussion sections to temper the interpretation of the sensory data.

The result section was revised as follows.

"Among the formulations, sPreF3 received numerically higher ratings for color, flavor, taste, and overall appearance; however, these differences were not statistically significant ($p > 0.05$). It was, nonetheless, significantly preferred for crunchiness compared to sPreF4 ($p < 0.05$). Overall, while sensory ratings were comparable across formulations, sPreF3 tended to score higher in most sensory attributes and was significantly preferred for crunchiness, suggesting a more desirable texture at this formulation level."

The discussion section was revised as follows.

"Sensory evaluations indicated that snack bars containing 40% pregelatinized GBF (sPreF3) received numerically higher ratings across most sensory attributes and were significantly preferred for crunchiness compared to sPreF4. This formulation demonstrated a favorable balance of texture, flavor, and appearance that likely contributed to its higher overall acceptability. Although differences in color, flavor, taste, and overall appearance were not statistically significant, sPreF3 consistently showed a favorable trend in sensory perception. This suggests that incorporating pregelatinized GBF at this concentration contributes to an optimal texture and a balanced sensory profile, likely due to suitable moisture management and desirable Maillard reaction products developed during baking. [44, 45]."

Figure 2 & 3: Figure 2: The labels (A-E) are missing from the figure itself. They must be added. Figure 3: The graphs (A, B, C) must include asterisks () or letters to indicate significant differences between the groups. The description in the legend is not a substitute for visual statistical annotation on the graph.

Address the reviewer's comment: Thank you for pointing this out. We apologize for the mistake. Labels (A-E) have now been added to Figure 2. Additionally, asterisks indicating the differences have been included in Figure 3, with further description provided in the legend.

Table 3 (Shelf Stability): As mentioned under "Inconsistencies," the microbiological data needs a brief comment in the results or discussion (e.g., "The low but detectable TVC after week 2 suggests minor post-processing contamination, but counts remained stable and within acceptable limits throughout the study."). The sensory scores show some significant fluctuations. This should be mentioned in the text, not just presented in the table.

Address the reviewer's comment: Thank you for this valuable comment. We have revised the Results and Discussion sections to temper the interpretation of the microbiological data and sensory data.

The result section was revised as follows.

"An acceptable microbiological quality was also achieved in the formulated snack bar, with total viable microbial counts stabilizing around 10^2 CFU/g after the second week. In comparison, yeast and mold counts remained generally low (<10 CFU/g). At week 0, total viable counts were below the detection limit (<10 CFU/g). The slight increase to approximately 10^2 CFU/g observed by week 2 may be attributed to minor post-baking contamination during handling or packaging, which is common in low-moisture bakery-type products. However, the microbial counts remained stable and well within acceptable limits for ready-to-eat snack products throughout the six-week storage period, indicating adequate hygienic handling and effective packaging integrity."

"Sensory evaluations consistently showed high acceptance scores, with minor fluctuations in color (moderately), crunchiness (from moderately to very much), flavor (from slightly to moderately), taste (from moderately to very much), and overall appearance (from moderately to very much). This suggests that storage may slightly influence consumer perception. Sensory evaluation scores showed statistically significant but minor fluctuations over the six-week period, particularly in crunchiness, flavor, and overall appearance. These changes may be related to gradual moisture absorption and minor shifts in texture or flavor perception over time. Nevertheless, the overall sensory acceptability remained high, confirming the product's stability and consumer appeal during storage."

DISCUSSION

Line 232-234: "This finding aligns with previous studies, which demonstrate that increased temperature during drying processes substantially boosts the swelling power and water solubility index of green bananas [33, 34]." – Excellent, situates your findings within existing literature.

Address the reviewer's comment: Thank you very much for your support and encouragement through your valuable comments. We sincerely appreciate it.

Line 255-257: "Sensory evaluations indicated that snack bars containing 40% pregelatinized GBF (sPreF3) exhibited the highest acceptability..." – As per the comment on Results, adjust this statement to accurately reflect the statistical results from Table 2.

Address the reviewer's comment: Thank you very much for bringing this to our attention. We have made the following revisions.

“Sensory evaluations indicated that snack bars containing 40% pregelatinized GBF (sPreF3) received numerically higher ratings across most sensory attributes and were significantly preferred for crunchiness compared to sPreF4.”

Line 270-271: "It should be highlighted that the pregelatinization process used in this study does not attenuate the gastroprotective properties of GBF." – This is a key conclusion and should be stated prominently.

Address the reviewer’s comment: Thank you for your valuable feedback. We have made the following revisions.

In summary, it should be highlighted that the pregelatinization process used in this study does not attenuate the gastroprotective properties of GBF.

Limitations Section (Line 295-301): This is well-written and strengthens the manuscript by showing scholarly awareness of the study's constraints.

Address the reviewer’s comment: Thank you very much for your support and encouragement through your valuable comments. We sincerely appreciate it.

CONCLUSION: Concise and accurately reflects the study's findings. No changes needed.

Address the reviewer’s comment: Thank you so much for your wonderful support and encouragement! It truly means a lot to our team

REFERENCES, FORMATTING, and TYPOGRAPHICAL ERRORS
Line 17 (Keywords): "peptic ulcers" is used here, but "gastric ulcers" is used in the title and throughout the text. For consistency and discoverability, consider using "gastric ulcers" as the keyword.

Address the reviewer’s comment: The keywords have been revised as follows:

“Gastroprotective effect; Pregelatinized green banana flour; Gastric ulcers; Functional food; Ethanol-induced gastric ulcer; Snack bar formulation”

Line 108: "sPreF3" should be defined here when first introduced (e.g., "snack bar made with PregelF3 (sPreF3)").

Address the reviewer’s comment: Thank you very much for bringing this to our attention. We have made the following revisions.

The lowest water activity and moisture content, at 0.327 and 3.63%, respectively, were observed in the formulated snack bar made with PregelF3 Flour (sPreF3).

General: Carefully proofread the manuscript for minor spacing and punctuation errors (e.g., "cm^2" sometimes appears as "cm2").

Address the reviewer's comment: Thank you for bringing this to our attention. We carefully proofread the manuscript, and all minor spacing and punctuation errors have been corrected.

This is a strong paper with a compelling story. The core experiments are valid, and the data supports the conclusions. The revisions required are primarily for clarity, statistical rigor, and accuracy rather than fundamental flaws in the research.

Address the reviewer's comment: Once again, we would like to express our gratitude to you. Thank you very much; we sincerely appreciate it.

Priority Actions:

1. Correct the major error in the Introduction regarding existing evidence for gastroprotective effects.

2. Clarify the methodology for administering the flour in the animal study.

3. Add statistical annotations (letters or asterisks) to all figures and tables where comparisons are made.

4. Ensure the narrative in the Results and Discussion sections accurately reflects the statistical outcomes, particularly for the sensory data. With these revisions, this manuscript will be significantly strengthened and should be considered for publication in a good journal.

Address the reviewer's comment: All comments have been addressed and clarified point by point. The changes and corrections have been made and highlighted in yellow in the marked revised manuscript.

Reviewer #2: The authors are advised to carefully address all the comments/suggestions/queries, I have made section-wise on the MS entitled "Formulation and In Vivo Evaluation of Unripe Banana Flour Snack Bars: A Promising Alternative for Medical Nutrition Therapy in Gastric Ulcers."

1. Title: The title needs to be improved and more accurate.

Address the reviewer's comment: Thank you very much for your feedback. The title was revised as follows.

"**Pregelatinized Green Banana Flour Snack Bars: Physicochemical Properties, Sensory Quality, and Gastroprotective Potential in an Ethanol-Induced Ulcer Model.** "

2. Abstract - The aim is very confused and needs to be improved. I don't understand this statement: "Including functional foods that support gastrointestinal health in the diet can aid in preventing peptic ulcers and minimizing their recurrence," as it can't be the beginning of the abstract. Change it. Also, the aim needs to be rewritten to be better understood. The abstract is missing numerical data on key findings. Keywords are general and vague. They need to be improved.

Address the reviewer's comment: Thank you very much for your suggestion. The abstract has been rewritten for clarity and detailed with the key findings' numerical data as follows. Additionally, the keywords have been corrected as mentioned.

"Peptic ulcers remain a recurrent gastrointestinal disorder requiring preventive nutritional strategies. Functional foods that promote gastrointestinal health may play an important role in preventing and managing peptic ulcers. In this study, we evaluated pregelatinized green banana flour (GBF) prepared by drum drying as a functional ingredient for developing snack bars with gastroprotective potential. The aim of this study was to systematically investigate how drum-drying pregelatinization influences the physicochemical and gastroprotective properties of GBF and to determine its suitability as a functional ingredient in the formulation of shelf-stable, consumer-acceptable snack bars. The pregelatinization process significantly reduced the moisture content and water activity, while improving water solubility index and swelling power. Snack bars formulated with 40 % pregelatinized GBF (sPreF3) showed the most desirable sensory profile, achieving high hedonic scores for crunchiness (7.470 ± 0.860) and overall appearance (7.40 ± 0.93) on a 9-point scale. During six weeks of storage at 25 °C and 50–70 % relative humidity, the product maintained stable moisture content (3.190 – 4.150 %), low microbial counts ($< 1.2 \times 10^2$ CFU/g), and consistent sensory acceptability. In vivo, both native GBF and pregelatinized GBF (1,000 mg/kg) significantly reduced ethanol-induced gastric ulcer area (0.020 ± 0.010 cm^2 and 0.090 ± 0.030 cm^2, respectively) compared with vehicle controls (0.410 ± 0.090 cm^2; $p < 0.05$), while increasing gastric mucin levels (26–27 µg/g tissue) and pH (4.5–5.0). This finding indicates that pregelatinized GBF exhibits significant gastroprotective benefits and excellent functional and sensory qualities, supporting its potential application in gastrointestinal health–promoting snack formulations. "

Keywords: Gastroprotective effect; Pregelatinized green banana flour; Gastric ulcers; Functional food; Ethanol-induced gastric ulcer; Snack bar formulation

3. Introduction

- The introduction is very short and scattered. It should cover all the points addressed in the study.
- Clear objective of the present work.

- The introduction should mention what is novel about this study.

Address the reviewer's comment: Thank you very much for your valuable comments. The introduction section has been rewritten for clarity as follows.

…"Among them, the unripe Musa species used to make green banana flour (GBF) can be singled out as a highly nutritious source of resistant starch, phenolic antioxidants, and non-digestible fiber [8-9]. Its gastroprotective properties have been demonstrated in several preclinical studies, which show reductions in gastric and intestinal inflammation across various models [10-15]. Moreover, its long-range ethnopharmacological history of use in traditional Thai and Sudanese medicine for the treatment of peptic ulcer disease has long been regarded as evidence of its therapeutic potential [13,16].

Nonetheless, there are challenges associated with the direct application of native GBF of food formulations. Its low solubility, roughness, and low digestibility limit its sensory properties and industrial application [17,18]."…

… "Although these advantages are well known, a research gap remains regarding the effects of pregelatinization on the gastroprotective activity of GBF and its functional properties in consumer-acceptable edible foods. The study was thus aimed at determining the physicochemical properties of pregelatinized drum-dried GBF, comparing its gastroprotective effects with those of native GBF using an ethanol-induced gastric ulcer rat model, and evaluating shelf-stable snack bars containing pregelatinized GBF in terms of their sensory and storage properties.

We posit that pregelatinization does not alter the gastroprotective performance of GBF but improves its functional and physicochemical characteristics, thereby developing a palatable, stable, health-promoting snack bar that can be used in gastrointestinal nutritional support."…

4. Methods: All the physicochemical analyses need to be divided into subheading titles to be more accurate and clear as they are very confused. The sensory evaluation needs to be in a separate section. What did you mean by this title: Gastroprotective effects? Moreover, this section is very confused, and the animal experiment needs to be organized as follows:

1. Experimental animals: Include animal details and ethical approval.
2. Laboratory diet composition: the authors need to mention a normal laboratory diet with references.
3. Study design: this section needs to be written in a scientific manner, as it is very confused, as the authors mentioned that there are two control groups: (1) sham control (distilled water); (2) vehicle control (distilled water); I don't know what the difference between them is, since both of them used distilled water. Also, the authors mentioned a positive control (omeprazole, 20 mg/kg). How is this positive control??? Omeprazole is a drug, and they used it to compare with the effect of using Unripe Banana Flour Snack Bars. Moreover, the positive control should be the group that had 80% ethanol (1 mL/rat).
4. Blood samples collection
5. Biochemical parameters: There are no biochemical parameters used in this study. The study should evaluate the activity of the treatment used in this study by measuring some biochemical parameters, including: Cytokines quantitation Tumor necrosis factor-α (TNF-α) and interleukins (IL-1, IL-6, IL-10, and IL-1β) should be measured using ELISA kits. Moreover, lipid peroxidation, oxidative stress markers, enzymatic and non enzymatic antioxidant levels, malondialdehyde (MDA) concentration, nitric oxide (NO) concentration, glutathione peroxidase (GPx), glutathione S-transferase (GST), glutathione reductase (GR), superoxide dismutase (SOD), and reduced glutathione (GSH) should be measured.
6. Statistical analysis- The authors need to compare all the treated groups to the positive and the drug control to prove their aim.

Address the reviewer's comment: Thank you very much for your valuable comments. The methods section has been rewritten point by point for clarity as follows.
1. The physicochemical analyses were organized into subheadings to improve accuracy and clarity.
2. The sensory evaluation was moved to a separate section.
3. The subtitle for the gastroprotective experiment was rewritten as "2.6. The gastroprotective effect of GBF and PregelF3 against ethanol-induced acute gastric lesions in rats".
4. The animal experiment has been rewritten and reorganized to include animal details and ethical approval information. It now also provides information about a standard laboratory diet, referencing the supplying company. Based on this, the commercial laboratory diet composition includes crude protein at 24%, fat at 4.5%, fiber at 5%, minerals (such as Ca 1.0%, Na 0.20%, K 1.17%, Mg 0.23%, Mn 171 ppm, Cu 22 ppm, Zn 100 ppm, Fe 180 ppm, Se 0.1 ppm), and vitamins (A 20,000 IU/kg, D 4,000 IU/kg, E 100 mg/kg, B1 5 mg/kg, B2 20 mg/kg, B6 20 mg/kg, B12 0.036 mg/kg, niacin 100 mg/kg, folic acid 6 mg/kg, biotin 0.4 mg/kg, pantothenic acid 60 mg/kg). Additionally, the Study Design section was rewritten to improve clarity for each group.
5. The additional information in the Study Design section was rewritten to improve clarity for each group. For more clarification of chosen omeprazole as a positive control. Omeprazole (20 mg/kg) was selected as the positive control because it is the clinical gold standard for treating gastric lesions. While its primary role is acid suppression, it is a well-established reference in ethanol-induced models due to its documented ability to enhance mucosal defensive factors (such as mucin production) and its antioxidant properties, which provide a robust benchmark for evaluating the gastroprotective potential of the Green Banana Flour (GBF) snack bars.
6. We thank the reviewer for the insightful comment on blood sample collection and biochemical evaluation. We agree that analyzing biochemical markers such as oxidative stress parameters (MDA, SOD) or inflammatory cytokines (TNF-α, IL-6) would provide a deeper understanding of the systemic antioxidant and anti-inflammatory mechanisms of the green banana flour snack bars.

However, due to practical and budget limitations at the time of the study, blood collection and systemic biochemical analysis were not performed. Instead, we focused our investigation on the primary physiological and morphological indicators of gastric mucosal integrity, which include (1) macroscopic ulcer area, the most direct measure of ethanol-induced damage, (2) histopathological examination, used to observe cellular-level protection and structural preservation, and (3) gastric pH and mucin content, used to evaluate the direct local defensive factors of the stomach. These parameters are well-established in the literature as definitive markers for assessing gastroprotective efficacy in ethanol-induced models. We have acknowledged this limitation in the revised manuscript.
6. Statistical analysis comparing all treated groups to the positive and drug control groups has been included in the revised manuscript.

5. Results: Make the order of the results section the same as you will make in the methods section.
Address the reviewer's comment: Thank you very much for your feedback. The results section in the revised manuscript has been carefully rewritten and organized in the same manner as the methods section.

6. Discussion: needs to be improved and written in scientific words.
Address the reviewer's comment: Thank you very much for your feedback. The discussion section of the revised manuscript has been carefully rewritten in scientific language as requested.

7. The conclusion needs to be rewritten and more advanced.
Address the reviewer's comment: Thank you for your feedback. The conclusion section was rewritten as requested.

"This study highlights the promising application of pregelatinized green banana flour (GBF), processed through drum drying, as a functional food ingredient with gastroprotective properties. This pregelatinization technique significantly improved the physicochemical properties of GBF, especially its water solubility and swelling capacity, thereby enhancing its functionality in food product development. Notably, this study confirms that drum-drying pregelatinization maintains the gastroprotective properties of green banana flour, yielding comparable efficacy to the native form in an ethanol-induced gastric ulcer model. This reinforces its suitability as a functional ingredient in medical nutrition formulations targeting gastrointestinal health. Furthermore, snack bars formulated with 40% pregelatinized GBF (sPreF3) exhibited the highest sensory acceptance, low water activity, and acceptable shelf life under ambient storage conditions. These results highlight the potential of drum-dried pregelatinized GBF as an economically viable, shelf-stable, and palatable vehicle for delivering gastrointestinal health benefits. In addition to its proven antiulcer activity, the modified GBF may have gut barrier effects, antioxidant protection, and microbiome regulation, which warrant further study with respect to its mechanistic impact. The molecular mechanisms of gastroprotective action of GBF, including but not limited to the control of oxidative stress, inflammatory mediators, and the mucin-secretory genes, need to be explored in future research, including the effectiveness of GBF in clinical or nutrigenomic settings. Increasing production and testing its applicability in other food matrices (e.g., beverages, cereals, or therapeutic diets) may also help to position GBF as a new type of nutraceutical ingredient in the functional food market. Together, this output offers a scientific ground in the application of pregelatinized GBF to medical nutrition therapy and peptic ulcer disease and gastrointestinal disease prevention measures."

8. References: Includes many old references and needs to be updated to be recent.
Address the reviewer's comment: Thank you for your feedback on the references section. The references section has been updated.

9. There are many grammatical errors, and the writing level is low and needs to be improved.
Address the reviewer's comment: Thank you for your feedback. The revised manuscript has been proofread by all authors to reduce grammatical errors and improve its readability. Furthermore, we have utilized Grammarly for an expert review to ensure there are no grammatical errors in the revised manuscript.

10. The authors need to organize this paper in a scientific manner and present their novelty.
Address the reviewer's comment: Thank you for your feedback and for encouraging our team to revise the manuscript. The revised manuscript was rewritten in a scientific manner and clearly highlights their novelty as suggested.
