# Supplementary material for: Pregelatinized green banana flour snack bars: Physicochemical properties, sensory quality, and gastroprotective potential in an ethanol-induced ulcer model
Source: PLoS One. 2026 Apr 24;21(4):e0347771. doi: 10.1371/journal.pone.0347771 (PMC13108814; doi:10.1371/journal.pone.0347771)
Supplement: Attachment — Submitted filename: Response to Reviewers.docx [file pone.0347771.s004.docx]

A detailed point-by-point response

| **Reviewer/Editor Comment** | **Author's Response and Action Taken** | **Location in Manuscript** |
| --- | --- | --- |
| 1. Grammar and Language |  |  |
| Line 44: "of food formulations" should be "in food formulations". | We have corrected the preposition as suggested. | Line 44 |
| Line 126: "pregelatinized GBF (pregelF1..." should be "pregelatinized GBF (PregelF1...". | We have corrected the capitalization to ensure consistency with defined terms. | Line 126 |
| Line 192: "...confirmed the model's sensitivity." Consider "confirmed the efficacy of the positive control and the model's sensitivity". | The sentence has been revised to clarify the role of the positive control and the model sensitivity. | Line 192 |
| Line 271: "Pregelatinized GBF developed in this present work serves..." -> "The pregelatinized GBF developed in this study serves...". | The text has been updated for better academic flow as suggested. | Line 271 |
| Conclusion Section: The conclusion is comprehensive but slightly verbose. Consider streamlining sentences.+1 | We have tightened the conclusion section, specifically splitting and streamlining the sentence regarding antiulcer activity to improve conciseness. | Conclusion section |
| 2. Figure and Table Consistency |  |  |
| Table 1: The decimal places are now consistent. | We have ensured that the consistency in decimal places is maintained throughout the revision. | Table 1 |
| Figure 2: Confirm that labels (A-E) are clearly visible in high-resolution files. | We confirm that labels A-E are clearly legible in the high-resolution figure files prepared for final submission. | Figure 2 |
| Table 3/4 Numbering: Ensure the numbering sequence is correct based on first mention. | We have re-checked and corrected the numbering sequence for Table 3 (*in vivo* effects) and Table 4 (shelf stability) to ensure they are sequential based on their first mention in the text. | Tables |
| 3. Funding Statement |  |  |
| Funding Statement (Lines 380-384): Ensure the statement includes the required disclaimer. | The funding statement remains clear and includes the mandatory disclaimer. | Lines 380-384 |
| 4. References |  |  |
| Please check all references and include recent citations from the last 5 years. | We have updated the reference list to include the suggested recent literature (DOIs: 10.1016/j.afres.2024.100431 and 10.1371/journal.pone.0307909) and verified the accuracy of all citations. | Reference List |
